# Supplementary material for: COVID-19 ARDS is characterized by a dysregulated host response that differs from cytokine storm and is modified by dexamethasone
Source: Res Sq. 2021 Jan 14:rs.3.rs-141578. Preprint. [Version 1] doi: 10.21203/rs.3.rs-141578/v1 (PMC7814832; doi:10.21203/rs.3.rs-141578/v1)
Supplement: 1 [file NIHPPrs141578v1-supplement-1.pdf]

Supplementary Materials

Supplementary Data 1. Detailed clinical, microbiological and demographic features of the cohort. Legend: *SaO_2/FiO_2 Ratio (SF Ratio) < 315 was used to verify ARDS diagnosis in these subjects.

Supplementary Data 2. Differentially expressed genes (adjusted P value (padj) < 0.1) between patients with ARDS due to COVID-19 (COVID-ARDS) versus **a)** controls with ARDS due to other etiologies (Other-ARDS) or **b)** intubated controls without ARDS (No-ARDS). Positive fold change indicates gene is upregulated in COVID-ARDS.

Supplementary Data 3. Ingenuity Pathway Analysis (IPA) of differentially expressed genes (padj < 0.1) between patients with ARDS due to COVID-19 (COVID-ARDS) versus **a)** controls with ARDS due to other etiologies (Other-ARDS) or **b)** intubated controls without ARDS (No-ARDS). Positive Z-score indicates pathway is upregulated in COVID-ARDS. Pathways with a Z-score absolute value ≥ 1 are included in table.

Supplementary Data 4. Plasma concentrations of inflammatory cytokines in patients with ARDS due to COVID-19 (COVID-ARDS) versus controls with ARDS due to other etiologies (Other-ARDS).

Supplementary Data 5. *In silico* deconvolution of cell type proportions from tracheal aspirate bulk RNA-sequencing data using lung single cell signatures. Data are plotted in (Supplementary Figure 1).

Supplementary Data 6. Chemical and biological drugs computationally predicted by IPA to attenuate the transcriptional response of ARDS due to COVID-19 (COVID-ARDS) against a comparator group of **a)** ARDS due to other causes (Other-ARDS) or **b)** intubated controls without ARDS (No-ARDS). Drugs with a Z-score > 2 are included in table.

Supplementary Data 7. Genes affected by drugs computationally predicted by IPA to modulate the transcriptional response of ARDS due to COVID-19 (COVID-ARDS) against a comparator group of **a)** ARDS due to other causes (Other-ARDS) or **b)** intubated controls without ARDS (No-ARDS). Predicted transcriptional effect of each drug on each gene is indicated with respect to comparator group 1 in the table.

Supplementary Data 8. Differentially expressed genes (padj < 0.1) between patients with ARDS due to COVID-19 (COVID-ARDS) versus controls with ARDS due to **a)** other viral lower respiratory tract infections (Other Viral-ARDS) or **b)** bacterial lower respiratory tract infections (Bacterial-ARDS). Positive $\log_2$ fold change indicates gene is upregulated in COVID-ARDS.

Supplementary Data 9. Pathway analysis (IPA) of differentially expressed genes (padj < 0.1) between patients with ARDS due to COVID-19 (COVID-ARDS) versus controls with ARDS due to other viral or bacterial lower respiratory tract infections. Z-scores are with respect to COVID-ARDS. Pathways with a Z-score absolute value ≥ 1 are included in table.

Supplementary Data 10. Computationally predicted (IPA) upstream cytokines based on the transcriptional signature of COVID-19 ARDS (COVID-ARDS) compared to ARDS from other viral LRTI. Pathways with a Z-score absolute value ≥ 1 are included in table.

Supplementary Data 11. Relationship between interferon-stimulated gene (ISG) expression and SARS-CoV-2 viral load measured in RNA-seq reads per million (rpM) for COVID-19 patients with late, severe disease and ARDS (COVID-ARDS) from lower respiratory tract samples (this study), and COVID-19 patients with early, mostly mild disease measured from upper respiratory tract samples[18]. Legend: reg_intercept = intercept in the robust regression of gene expression on SARS-CoV-2 viral load; reg_slope = slope in the robust regression of gene expression on SARS-CoV-2 viral load; reg_adj_R2 = adjusted robust coefficient of determination; reg_p_adj = Benjamini-Hochberg adjusted p-value for difference of the regression slope from 0.

Supplementary Appendix. COMET Consortium member list.

Supplementary Figure 1. *In silico* deconvolution of cell types from tracheal aspirate bulk RNA-sequencing data using lung single cell signatures. The horizontal line inside the box denotes the median and the lower and upper hinges correspond to the first and third quartiles, respectively. Whiskers extend from the hinge to the largest (smallest, respectively) value no more than 1.5*IQR away from the hinge, where IQR is the interquartile range. The y-axis in each panel was trimmed at the maximum value among the three patient groups of 1.5*IQR above the third quartile. Pairwise comparisons between patient groups were performed with a two-sided Mann-Whitney-Wilcoxon test followed by Bonferroni's correction (n=15 COVID-ARDS, n=32 Other ARDS, n=5 No-ARDS). Data are tabulated in (Supplementary Data 5).

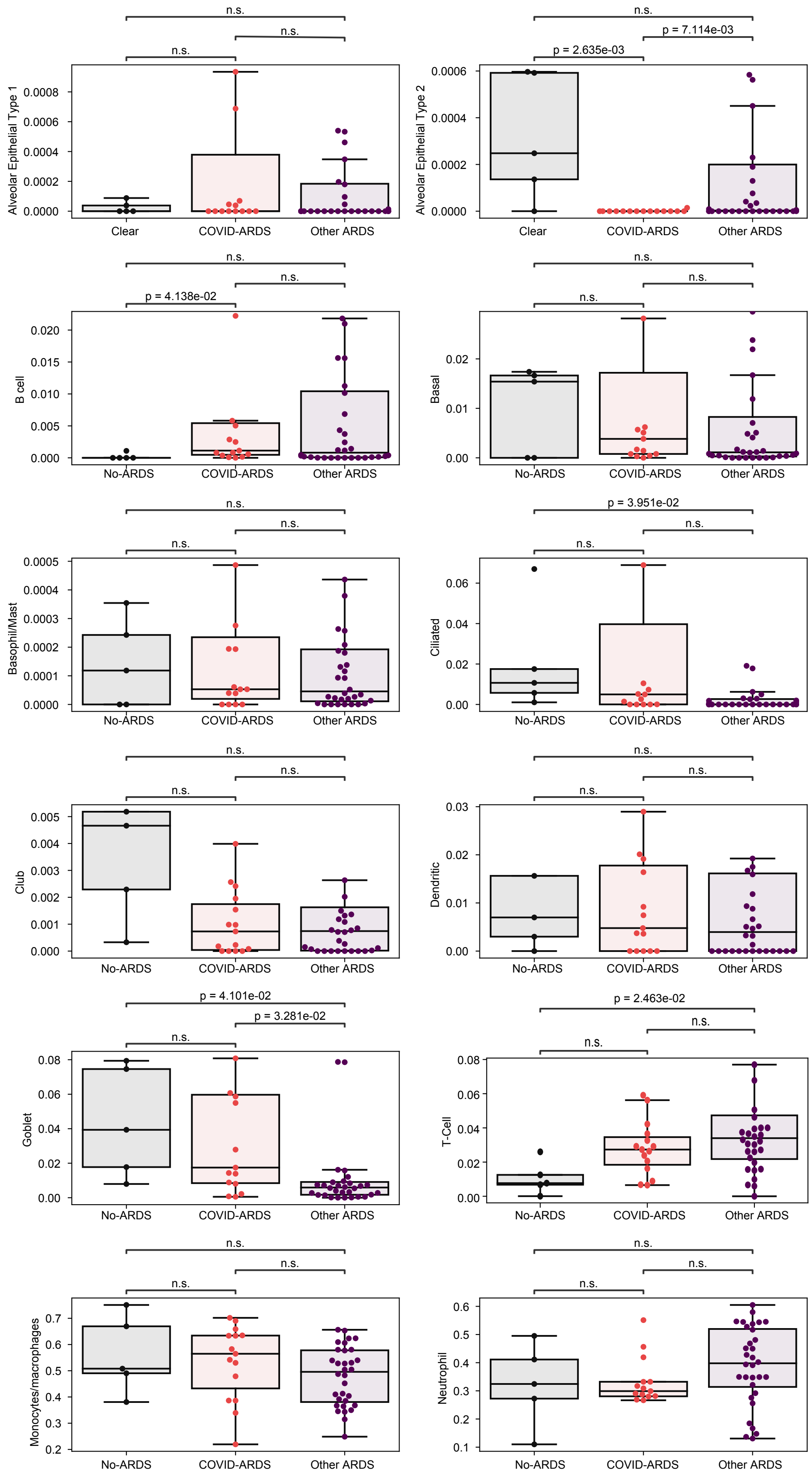
n.s.
n.s.
n.s.
Alveolar Epithelial Type 1
Clear
COVID-ARDS
Other ARDS
n.s.
p = 7.114e-03
p = 2.635e-03
Alveolar Epithelial Type 2
B cell
p = 4.138e-02
No-ARDS
Basal
Basophil/Mast
p = 3.951e-02
Ciliated
Club
Dendritic
p = 4.101e-02
p = 3.281e-02
Goblet
p = 2.463e-02
T-Cell
Monocytes/macrophages
Neutrophil

Figure S1
